# Neuropathie optique bilatérale induite par un traitement antibacillaire chez un patient insuffisant rénal

**DOI:** 10.11604/pamj.2013.16.42.3378

**Published:** 2013-10-06

**Authors:** Hanan Handor, Wafae Ibrahimy

**Affiliations:** 1 Université Mohammed V Souissi, Service d'Ophtalmologie A de l'hôpital des spécialités, Centre hospitalier universitaire, Rabat, Maroc

**Keywords:** Neuropathie optique, traitement antibacillaire, insuffisance rénale, ethambutol, optic neuropathy, antibacillary treatment, renal failure, ethambutol

## Image en médecine

La tuberculose est une maladie infectieuse devenue actuellement curable grâce à un traitement médical bien conduit. Malgré leur grande efficacité, les antibacillaires peuvent être responsables d'effets secondaires potentiellement graves nécessitant une surveillance rigoureuse au cours du traitement. Nous rapportons un cas de neuropathie optique bilatérale induite par des antibacillaires chez un patient ayant une insuffisance rénale au stade terminal. Il s'agit d'un patient de 42 ans, hémodialysé chronique depuis 11 ans pour une néphropathie au stade d'insuffisance rénale, qui s'est présenté aux urgences pour baisse bilatérale de l'acuité visuelle associé à un scotome central. L'interrogatoire a retrouvé la notion de traitement antibacillaire (Ethambutol, Isoniazide, Rifampicine et Pyrazinamide) démarré il y a 1 mois et demi pour traitement d'une tuberculose pulmonaire. L'anamnèse a retrouvé également une notion d'hypoacousie et de fourmillements des membres supérieurs. L'examen ophtalmologique a révélé une acuité visuelle réduite à 1/10 au niveau des deux yeux, avec au fond d’œil un œdème papillaire bilatéral. Les explorations para cliniques, notamment la neuro imagerie, le champ visuel, la vision des couleurs et les potentiels évoqués visuels ont permis de retenir le diagnostic de neuropathie optique bilatérale liée à la toxicité du traitement antibacillaire. L'ethambutol a été arrêté et le malade a été mis sous vitaminothérapie. L’évolution a été marquée par une légère amélioration de l'acuité visuelle qui est remontée à 2/10 au niveau des deux yeux. A travers cette observation, nous insistons sur le risque de neuropathie optique induite par le traitement antibacillaire, particulièrement par l'ethambutol et l'isoniazide. La surveillance ophtalmologique durant toute la période du traitement est impérative et l'adaptation des posologies aux patients insuffisants rénaux est nécessaire.

**Figure 1:**
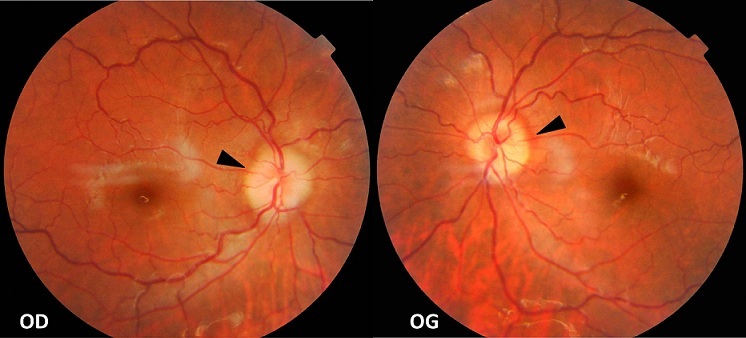
Image du fond d’œil objectivant l'aspect flou des bords des deux papilles traduisant un œdème papillaire bilatéral

